# Evaluation of Bioactivity and Biochemical Composition of *Galium aparine* L.

**DOI:** 10.3390/microorganisms14040804

**Published:** 2026-04-01

**Authors:** Kerem Canlı, Dilay Turu, Atakan Benek, Aslı Sade Memişoğlu, Aydın Çömez, Arzu Yücel, Hafize Handan Öner, Mustafa Burak Arslan, Gamze Gürsu, Fatma Duygu Özel Demiralp, Pınar Akan, Cevher Gündoğdu Hızlıateş, Görkem Akıncı

**Affiliations:** 1 Department of Biology, Faculty of Science, Dokuz Eylül University, Izmir 35390, Türkiye; 2 Fauna and Flora Research and Application Center, Dokuz Eylül University, Izmir 35390, Türkiye; 3 Department of Biology, Graduate School of Natural and Applied Science, Dokuz Eylül University, Izmir 35390, Türkiye; 4 Department of Science Education, Department of Mathematics and Science Teaching, Buca Faculty of Education, Dokuz Eylul University, Izmir 35340, Türkiye; 5 General Directorate of Forestry, Ege Forestry Research Institute, İzmir 35530, Türkiye; 6 Department of Basic Sciences, Faculty of Engineering and Natural Sciences, Maltepe University, Istanbul 34857, Türkiye; 7 Environment and Energy Technologies Research Center, Maltepe University, Istanbul 34857, Türkiye; 8 Türkiye Biotechnology Institute, Mamak, Ankara 06270, Türkiye; 9 Department of Medical Biochemistry, Faculty of Medicine, Dokuz Eylul University, Izmir 35340, Türkiye; 10 Department of Chemistry, Faculty of Science, Dokuz Eylul University, Izmir 35160, Turkey; 11 Science and Technology Application and Research Center, Dokuz Eylül University, İzmir 35390, Türkiye

**Keywords:** *Galium aparine* L., antioxidant activity, chemical composition, antibiofilm, quorum sensing

## Abstract

Due to increasing antimicrobial resistance and oxidative stress, the exploration of new therapeutic agents from medicinal plants such as *Galium aparine* L., has become essential. This study aimed to provide a scientific basis for the ethnomedicinal applications of *G. aparine* by evaluating its biological activities and biochemical composition. Plant samples were extracted using different solvents and their biological activities were assessed through antimicrobial, antibiofilm, and anti-quorum sensing (AQS) assays against different microorganisms. Antioxidant capacity was determined using four complementary assays representing distinct mechanisms, while the biochemical composition was analyzed via GC–MS. The results revealed that although the extracts exhibited limited antimicrobial activity overall, significant inhibition was observed against *Staphylococcus aureus*, and *Enterococcus* strains, with MIC values as low as 30 µg/mL. The ethanol extract demonstrated the highest antibiofilm inhibition (60.38%) against *Streptococcus mutans*, surpassing the positive control Halamid, and showed the strongest AQS inhibition (46.16%). Moreover, a strong antioxidant potential comparable to ascorbic acid was detected in the ABTS assay, with 88.10% inhibition. Overall, these findings indicate that *G. aparine* extracts possess notable antibiofilm, AQS, and antioxidant properties that support their traditional medicinal uses and suggest their potential as promising sources for the development of new natural therapeutic agents.

## 1. Introduction

Bacteria colonize diverse human tissues, including the gastrointestinal tract and skin, and constitute a core component of the human microbiota [1,2]. While the majority of bacterial species are harmless—and some even play crucial roles in digestion and in limiting the growth of opportunistic pathogens—bacterial infections remain among the most common causes of disease worldwide [3,4]. Over recent decades, the alarming increase in antimicrobial resistance (AMR) has emerged as a major global health crisis, significantly complicating the treatment of infections caused by resistant strains [5,6]. The selection of effective antibiotics has become increasingly difficult [5], and although new antimicrobial agents have been introduced in recent years [5], resistance to many of these therapies has already been reported [5,7]. The rise in extensively drug-resistant (XDR) pathogens has diminished the efficacy of even the most potent antibiotics, highlighting the urgent need for novel therapeutic strategies and alternative treatment approaches [8,9].

In addition to AMR, oxidative stress represents another critical factor threatening human health. Oxidative stress occurs when reactive oxygen species (ROS), produced as by-products of cellular metabolism, exceed the body’s antioxidant defense capacity, leading to molecular and cellular damage [10]. The accumulation of ROS has been strongly linked to the pathogenesis of numerous chronic diseases, including cancer, metabolic syndrome, atherosclerosis, malaria, Alzheimer’s disease, rheumatoid arthritis, neurodegenerative disorders, and preeclampsia [11]. Antioxidants, which neutralize ROS and free radicals, are broadly classified as natural or synthetic compounds [12]. Among these, plant-derived natural antioxidants are of particular interest, as their bioactivity is influenced by factors such as plant species, cultivation conditions, and extraction or processing techniques [13]. Growing evidence indicates that natural compounds not only reduce oxidative stress but may also enhance immune system function [14]. Thus, identifying plant-based bioactive molecules with antioxidant and antimicrobial potential has become a priority in biomedical research [15].

Medicinal plants have been utilized as a source of therapeutic agents for thousands of years, and it is estimated that 25–48% of currently available pharmaceuticals are derived from plants or their synthetic analogs, reflecting a growing interest in botanical resources [16]. The genus *Galium*, a member of the Rubiaceae family, comprises roughly 1000 species globally and is represented by 101 species in Turkey [17]. *Galium aparine* L. (Cleavers) is an annual herbaceous plant. It flowers between April and July and occurs at altitudes ranging from 30 to 1800 m, typically in weeds, shrubs, and cultivated fields [18]. Ethnobotanical records indicate that the entire plant—including stems, leaves, flowers, and seeds—has long been used in folk medicine as a febrifuge, diuretic, and blood purifier to promote lymphatic circulation, and for the treatment of urinary tract infections, skin disorders such as eczema and psoriasis, ulcers, and chronic wounds. It has also been applied to alleviate swelling, infection, and inflammation, as well as to control bleeding caused by injuries [19].

Previous phytochemical investigations on *Galium aparine* have identified a diverse array of bioactive constituents, including phenolic acids (such as caffeic and p-coumaric acids), iridoids (notably asperuloside and aucubin), and various flavonoid glycosides. These compounds have been associated with a range of biological properties, particularly antioxidant and antimicrobial activities, as documented in several regional studies. While the general antimicrobial potential of *G. aparine* has been explored, most prior research has focused on crude aqueous or methanolic extracts against common pathogens. However, there remains a significant gap in the literature regarding a systematic comparison of extraction solvents on its chemical profile and, more importantly, its role in disrupting bacterial communication. To the best of our knowledge, this study represents the first comprehensive report on the Anti-Quorum Sensing (AQS) activity of *G. aparine*, complemented by a detailed GC-MS profiling of its ethanolic extract. By targeting the coordination of bacterial virulence rather than mere growth inhibition, this research provides a novel perspective on the therapeutic potential of this species as a sophisticated anti-virulence agent [20,21].

Beyond traditional bactericidal approaches, targeting bacterial virulence factors has emerged as a promising strategy to combat the global rise in antimicrobial resistance. A critical component of bacterial pathogenicity is the formation of biofilms, complex multicellular communities encased in a self-produced extracellular polymeric substance (EPS) matrix. Biofilms play a pivotal role in chronic infections by providing a physical and metabolic barrier that renders bacteria up to 1000-fold more resistant to conventional antibiotics and host immune responses. The developmental transition from a planktonic state to a biofilm is largely regulated by Quorum Sensing (QS), a sophisticated cell-to-cell communication system based on the secretion and detection of signaling molecules. Since QS coordinates the expression of virulence genes and stabilizes the biofilm architecture, disrupting these pathways—often referred to as an anti-virulence strategy—offers a potent means to attenuate bacterial pathogenicity without exerting the selective pressure that typically leads to multi-drug resistance. Consequently, evaluating the inhibitory potential of natural products like *Galium aparine* against these mechanisms is essential for developing novel therapeutic alternatives [22].

The selection of extraction solvents with varying polarities—ranging from non-polar n-hexane to highly polar methanol and ethanol—is crucial for a comprehensive phytochemical evaluation, as the solubility and recovery of bioactive secondary metabolites are strictly dependent on the solvent’s dielectric constant. Despite the documented traditional uses of *G. aparine*, a significant knowledge gap persists regarding its specific role in modulating bacterial social behaviors. To date, limited information exists regarding the antibiofilm and anti-quorum sensing potential of *G. aparine* extracts, and a comprehensive biochemical characterization in correlation with these advanced bioactivities remains scarce. This study is necessary now to address the urgent need for non-biocidal anti-virulence agents in the face of escalating antibiotic resistance. By systematically evaluating the influence of solvent polarity on the extraction of bioactive metabolites and linking these chemical profiles to the disruption of biofilm and quorum sensing pathways, this research aims to fill this critical void. Consequently, our findings provide a novel scientific rationale for the potential use of *G. aparine* as a sustainable and effective candidate for the development of next-generation anti-infective therapies.

## 2. Materials and Methods

### 2.1. Plant Sample

Within the scope of field studies conducted in April 2023, specimens of *G. aparine* were collected from diverse ecological habitats in the Aegean Region of Türkiye. Sampling was performed on 11 April 2023, in the mountainous areas of Kavacık village, the Huzur region, and Narlıdere, specifically from understories of Turkish red pine (*Pinus brutia*) and black pine (*Pinus nigra*). Additional surveys were carried out on 17 April 2023, in the Bozdağ region, Salihli, and Bayındır. Detailed site characteristics were recorded, including a Turkish red pine stand in Salihli–Allahdiyen (660 m altitude; 38°26′13″ N–28°05′22″ E) and an oak–Turkish red pine habitat in Bayındır (560 m altitude; 38°16′29″ N–27°39′14″ E).

The botanical authentication of the species was performed by expert biologists within the research team. A voucher specimen has been deposited at the Fauna and Flora Research and Application Center of Dokuz Eylül University with the herbarium number GA-3044. The whole plant (including aerial parts and roots) was used for the extraction process. To account for environmental variability and ensure a representative chemical profile, samples from different sites were pooled to form a composite sample. The plant materials were dried in a cool, shaded environment for three days until a constant weight was achieved and subsequently stored under controlled conditions until further analysis.

### 2.2. Sample Extraction Method

For the extraction of plant samples, ethanol, methanol, n-hexane, acetone, and water were selected as solvents. The choice of solvents was based on their ability to extract the most effective bioactive compounds, taking into consideration the solvent-specific extraction capacities reported by Cowan [23]. Since distilled water was also evaluated as a separate extraction solvent, absolute ethanol was preferred to avoid the influence of water in the ethanolic extraction system and to allow a clearer comparison among solvents of different polarities. For each extraction, 20 g of dried whole plant material was placed into individual Erlenmeyer flasks, to which 200 mL of distilled water, ethanol (99.9% purity), methanol (99.9% purity), acetone (≥99.8% purity), or n-hexane (≥99.0% purity) was added. The flasks were placed on an orbital shaker at room temperature and agitated at 160 rpm for three days. At the end of the third day, the mixtures were filtered through Whatman No. 1 filter paper. The solvents in the filtrates were evaporated using a rotary evaporator at 35–40 °C. The remaining crude extracts were weighed with an analytical balance. Following quantification, an appropriate volume of solvent was added to dissolve the dried residues, and the extracts were prepared [24].

For the aqueous extract, after filtration, the samples were frozen completely at −80 °C and subsequently lyophilized to remove water. The dried residues were then weighed with an analytical balance, and distilled water was added to prepare the extracts. To ensure sterility, all extracts were passed through a 0.45 μm pore-size membrane filter prior to biological assays. For experiments conducted without the use of organic solvents, Dimethyl sulfoxide (DMSO) was employed. To minimize toxicity to microorganisms, extracts dissolved in DMSO were diluted with sterile distilled water to a final concentration of 1% DMSO.

The resulting dry extracts were reconstituted in their respective extraction solvents and subsequently diluted in a DMSO:Water (1:99, *v*/*v*) mixture to prepare high-concentration working stocks for biological assays. While the percentage yield was not calculated, the precise stock concentrations were determined as follows: 20.94 mg/mL for ethanol, 39.84 mg/mL for methanol, 3.97 mg/mL for acetone, and 9.11 mg/mL for n-hexane. Their corresponding final working concentrations in the DMSO:Water system were adjusted to 76.4, 276.4, 15.3, and 63.4 mg/mL, respectively. Regarding storage, the prepared extracts were kept in light-protected, airtight glass vials at 4 °C for short-term use (less than 7 days) and at −20 °C for long-term storage to maintain the stability of bioactive constituents until the completion of the bioassays.

### 2.3. Microorganisms and Inoculum Preparation

To evaluate the antimicrobial activities of the obtained extracts, 45 different microorganisms were selected. The study aimed to provide a comparative assessment of the extracts by maintaining a broad spectrum of test organisms. The microorganisms were obtained from the Microbiology Laboratory, Department of Biology, Faculty of Science, Dokuz Eylül University. The panel included Gram-positive bacteria, Gram-negative bacteria, and yeasts. Among the tested microorganisms, 16 were standard strains, 7 were foodborne, 11 were antibiotic-resistant strains, and 11 were clinical isolates.

The standard strains were *Acinetobacter baumannii* CECT 9111, *Bacillus subtilis* DSM 1971, *Bacillus cereus* RSKK 863, *Enterobacter aerogenes* ATCC 13048, *Enterococcus faecalis* ATCC 29212, *Escherichia coli* ATCC 25922, *Listeria monocytogenes* ATCC 7644, *Pseudomonas aeruginosa* DSM 50071, *Pseudomonas fluorescens* P1, *Salmonella enteritidis* ATCC 13076, *Salmonella typhimurium* SL1344, *Staphylococcus aureus* ATCC 25923, *Staphylococcus epidermidis* DSM 20044, *Staphylococcus hominis* ATCC 27844, *Staphylococcus warneri* ATCC 27836, and *Shigella flexneri* RSKK 184.

The foodborne isolates were *Enterococcus durans*, *Enterococcus faecium*, *Escherichia coli*, *Klebsiella pneumoniae*, *Listeria innocua*, *Salmonella infantis*, and *Salmonella kentucky*. The antibiotic-resistant strains were *Achromobacter* sp., *Acinetobacter baumannii*, *Enterobacter aerogenes*, *Escherichia coli*, *Klebsiella pneumoniae*, *Proteus vulgaris*, *Providencia rustigianii*, *Serratia odorifera*, *Staphylococcus aureus* MRSA, *Staphylococcus aureus* MRSA 2, and *Streptococcus pneumoniae*. The clinical isolates were *Acinetobacter baumannii*, *Enterococcus faecalis*, *Klebsiella pneumoniae*, *Shigella boydii*, *Shigella flexneri*, *Staphylococcus aureus*, *Staphylococcus aureus* 2, *Staphylococcus haemolyticus*, *Staphylococcus hominis*, *Staphylococcus lugdunensis*, and *Streptococcus mutans*.

Bacterial cultures were incubated at 37 °C for 24 h. Inoculum densities were normalized to approximately 10^8^ cfu·mL^−1^ for bacterial strains by adjusting suspensions to the 0.5 McFarland standard in 0.9% sodium chloride solution [25].

### 2.4. Antimicrobial Activity Tests

#### 2.4.1. Disc Diffusion Method

The antimicrobial activity of *G. aparine* extract was evaluated using the disk diffusion method [26]. Sterile 6 mm blank paper disks were impregnated with standardized volumes of the extracts (50, 100, and 150 µL), corresponding to specific mass concentrations: 1.05–3.15 mg for ethanol, 2–6 mg for methanol, 0.45–1.35 mg for n-hexane, and 0.20–0.60 mg for acetone extracts. To ensure methodological standardization, the procedures were conducted in accordance with the general principles of the Clinical and Laboratory Standards Institute (CLSI) guidelines [27,28]. Disks loaded only with the extraction solvents served as negative controls to verify that the observed inhibition zones were solely due to the plant metabolites and not the residual solvent. All inhibition zones were measured in millimeters (mm) following the designated incubation period. Bacterial strains were incubated at 37 °C for 24 h. Gentamicin and clindamycin were used (Appendix A) as positive controls for Gram-negative bacteria and Gram-positive bacteria, respectively.

#### 2.4.2. Minimum Inhibitory Concentration (MIC) Assay

The broth microdilution method, as outlined by Kowalska–Krochmal and Dudek–Wicher [29], was used to determine the minimum inhibitory concentration (MIC) in order to evaluate the antibacterial properties of the *G. aparine* DMSO extracts. For MIC determination, the dried extracts were tested at maximum concentrations of 36,800 µg/mL for the ethanol extract, 92,130 µg/mL for the methanol extract, 5100 µg/mL for the acetone extract, and 21,130 µg/mL for the n-hexane extract, and serial dilutions were prepared in Mueller–Hinton broth. Different microbial strains were first adjusted to the turbidity of a 0.5 McFarland standard (approximately 1.5 × 10^8^ CFU/mL) and then diluted in Mueller–Hinton broth to obtain a final inoculum of approximately 5 × 10^5^ CFU/mL in each well, in accordance with CLSI recommendations [27]. A variety of plant extract dilutions were made and added to a 96-well plate, with 100 µL going into each well. The final volume was then increased to 150 µL by adding 50 µL of the microbial solution. Following a 24 h incubation, the MIC—the lowest concentration of the extract that fully inhibited bacterial growth—was determined through visual examination. To ensure accuracy, all tests were conducted in triplicate, and results were given in µg/mL.

#### 2.4.3. Minimum Bactericidal Concentration (MBC) Assay

The Minimum Bactericidal Concentration (MBC) approach was used to find the lowest concentration of an antibiotic capable of killing a pathogenic bacterium. This assay was conducted following the MIC test. From the wells in which no visible growth was observed in the MIC assay, 10 µL was taken and streaked onto MHA plates. The inoculated bacteria were incubated at 37 °C for 24 h. The lowest concentration at which no growth was observed after incubation was defined as the MBC [30]. To distinguish between bacteriostatic and bactericidal effects, the MBC/MIC ratio was calculated. The extract was defined as bactericidal when the MBC/MIC ≤ 4 and bacteriostatic when the MBC/MIC > 4.

#### 2.4.4. Antibiofilm Activity Test

The antibiofilm assays were performed in 96-well polystyrene microplates. To ensure the accuracy of the results, all absorbance readings were corrected using blank wells containing only the medium and the respective extract without microbial inoculation to account for any background interference. The determination of the antibiofilm activity of plant extracts was carried out in two stages: (i) the assessment of biofilm-forming capacity and (ii) the evaluation of antibiofilm activity. In the first stage, LB Broth (Lysogeny Broth, Luria–Bertani) was dispensed into sterilized glass test tubes, and glucose monohydrate was added at six different concentrations (0%, 0.5%, 1%, 1.5%, 2%, and 2.5%). The media were subsequently re-sterilized by autoclaving. For staining the biofilms formed in microplate wells, a 0.1% crystal violet solution prepared in ethanol was used. To quantify the amount of crystal violet absorbed by the biofilms, a 3:7 acetone:ethanol mixture was applied [31]. For biofilm formation assays, bacterial suspensions were adjusted to 0.5 McFarland and diluted in the corresponding broth medium to obtain an initial inoculum of approximately 1 × 10^6^ CFU/mL in each well.

The bacterial strains were incubated at 37 °C and the yeasts at 27 °C for 24 or 48 h. After incubation, the microplate wells’ contents were disposed of, and planktonic cells were eliminated by washing the wells with distilled water. After drying, 200 μL of crystal violet solution was added to each well and allowed to stand for 15 min. The wells were then washed again, left to dry, and subsequently treated with an ethanol–acetone solution for 15 min. The absorbance values of the released dye were measured spectrophotometrically, enabling the determination of the appropriate glucose concentration and incubation time to be used in further assays. All experiments in this stage were performed in triplicate, with LB broth without any additives serving as the negative control [32].

In the second stage, the experiment was repeated under the same conditions, with the only modification being the addition of plant extracts at ½ MIC concentrations prior to inoculation. In both the biofilm formation and antibiofilm activity assays, ethanol–acetone solutions from the treated wells were transferred to fresh microplates, and absorbance values were recorded at 550 nm using a microplate reader. All assays were carried out in triplicate. Halamid was used as the positive control, while LB broth without any additives served as the negative control. The percentage of biofilm inhibition was calculated using the following formula:

Biofilm Inhibition (%) = [1 − (ODsample/ODcontrol)] × 100

#### 2.4.5. Antiquorum Sensing Activity Test

Pyocyanin is a blue phenazine derivative characteristic of *Pseudomonas aeruginosa*. Upon acidification, its color changes first to yellow and then to red, while it becomes colorless under alkaline conditions. It is soluble in both water and chloroform [33,34]. The anti-quorum sensing activity was evaluated using the *P. aeruginosa* PA01 strain. To ensure that the observed reduction in pyocyanin production was specifically due to the disruption of quorum sensing pathways and not a consequence of growth suppression, bacterial growth was monitored by measuring the optical density at 600 nm (OD_600_) throughout the incubation period. Only extracts that did not significantly inhibit planktonic growth at the tested concentrations were evaluated for pyocyanin inhibition. To assess the inhibitory effect of plant extracts on pyocyanin pigment production, 100 μL of the extract was added to LB broth along with a bacterial culture adjusted to OD_600_ = 0.05, followed by incubation at 37 °C with shaking for 9 h. Subsequently, the cultures were further incubated with shaking at 37 °C for 24 h. At the end of the incubation period, 5 mL of chloroform was added to the culture medium and vortexed for 30 s. The separated chloroform phase (2 mL) was transferred into glass tubes, to which 1 mL of an HCl–H_2_O mixture (0.2 mmol^−1^) was added and vortexed for 30 s. The resulting pink phase formed at the top of the tubes was measured spectrophotometrically at 520 nm. All experiments were conducted in triplicate. The extract solvent served as the negative control [35,36]. The percentage of pyocyanin inhibition was calculated using the following formula:

Pyocyanin Inhibition (%) = [1 − (OD_520_(sample)/OD_600_(sample))/(OD_520_(control)/OD_600_(control))] × 100

### 2.5. Antioxidant Activity Test

The antioxidant potential of the *G. aparine* ethanol extract was evaluated across a concentration range of 0.00781 to 1 mg/mL using for 2,2-diphenyl-1-picrylhydrazyl (DPPH). For the DPPH assay, the IC50 values were calculated using a four-parameter log-logistic (4PL) non-linear regression model to ensure precise estimation of the dose–response relationship. The results for DPPH and ABTS were expressed as percentage inhibition, while the reducing capacities in CUPRAC and FRAP assays were quantified as Ascorbic Acid Equivalents (AAE) based on standardized calibration curves. Procedural descriptions followed established protocols.

#### 2.5.1. 2,2-Diphenyl-1-picrylhydrazyl (DPPH) Assay

The antioxidant potential of *G. aparine* was determined through the DPPH free radical scavenging assay. This widely applied method assesses the capability of antioxidant molecules present in plant extracts to quench stable free radicals. For the preparation of the working solution, DPPH was dissolved in ethanol and kept in a dark environment to maintain its stability and prevent photodecomposition [37]. Subsequently, different concentrations of the extracts were mixed with the DPPH solution in a 96-well microplate. After incubation for 30 min at ambient temperature under dark conditions, the absorbance was recorded at 515 nm using a Microplate Spectrophotometer (Biotek, Winooski, VT, USA). Ascorbic acid was utilized as a reference antioxidant for comparison purposes.

#### 2.5.2. Ferric Reducing Antioxidant Power (FRAP) Assay

The ferric reducing antioxidant power (FRAP) of *G. aparine* extract was determined according to the protocol described by Benzie et al. [38], with minor adjustments. Three stock solutions were prepared: acetate buffer, TPTZ solution, and FeCl_3_·6H_2_O. In the assay procedure, 2.9 mL of the reagent was mixed with 0.1 mL of the plant extract, resulting in the formation of an intense blue complex due to the reduction of Fe^3+^ to Fe^2+^. The reaction mixtures were kept at room temperature in darkness for 90 min before recording the absorbance at 593 nm. All analyses were conducted in triplicate. Ascorbic acid served as the standard antioxidant reference, while the solvent mixed with FRAP reagent functioned as the negative control.

#### 2.5.3. Cupric Reducing Antioxidant Capacity (CUPRAC) Assay

The cupric reducing antioxidant capacity (CUPRAC) of the extracts was evaluated based on the method proposed by Apak et al. [39], with slight modifications. The stock solutions included CuCl_2_·2H_2_O, neocuproine in methanol, and ammonium acetate buffer. For each assay, equal volumes (1 mL) of CuCl_2_·2H_2_O, neocuproine, and ammonium acetate buffer were combined with 1 mL of the plant extract. The reaction mixtures were then incubated for 90 min at room temperature in the dark. The appearance of a yellow color indicated the reduction of Cu(II) to Cu(I). Absorbance was subsequently recorded at 450 nm. All determinations were performed in triplicate. Ascorbic acid was used as the positive control, whereas the extraction solvent alone was used as the negative control.

#### 2.5.4. 2,2′-Azino-bis(3-ethylbenzothiazoline-6-sulfonic acid) (ABTS) Assay

The ABTS radical scavenging capacity was assessed following the procedures outlined by Miller and Rice-Evans [40] and Re et al. [41]. The ABTS•^+^ radical cation was produced by reacting ABTS solution with potassium persulfate in equal volumes (1:1, *v*/*v*), followed by incubation in the dark for 12–16 h. The resulting solution was diluted with methanol to obtain an absorbance of 0.700 ± 0.05 at 734 nm. For the assay, 2 mL of the ABTS•^+^ working solution was mixed with 1 mL of the plant extract, and the mixture was incubated for 90 min at room temperature. Absorbance readings were then recorded at 734 nm. Each test was performed in triplicate, with ascorbic acid serving as positive control and methanol as the negative control.

### 2.6. Gas Chromatography–Mass Spectroscopy Method (GC-MS)

Gas Chromatography–Mass Spectrometry (GC-MS) was employed to identify and quantify the biochemical constituents present in the ethanol, methanol, *n*-hexane and acetone extracts of *G. aparine*. The GC-MS analyses were carried out using Agilent GC 8890 and Agilent GC/MSD 5977B with HP-5 MS column (Santa Clara, CA, USA). The analytical procedure and instrumental parameters adhered to the methodology established by Simsek et al. [42]. The mass spectrometer was operated in positive electron impact (EI+) mode at 70 eV ionization energy, with a scan range of 42–650 *m/z* and a cycle time of 0.5 s. The MS transfer line, interface, and ion source temperatures were maintained at 280 °C, 280 °C, and 230 °C, respectively. All peaks were quantified based on peak area. Compound identification was performed by matching the observed retention times (RT) and MS fragmentation patterns with those in the Wiley-Nist MS library and established literature data (Hoboken, NJ, USA).

### 2.7. Statistics

Statistical analyses were conducted using R Studio (2025.09.1). To ensure data reliability, antimicrobial activity and all other biological assays were performed as three independent experiments, each containing internal technical replicates. The Shapiro–Wilk test was used to verify the normality of the data, and Levene’s test was applied to assess the homogeneity of variances. Differences between experimental groups (solvents and concentrations) were evaluated using one-way or two-way ANOVA, followed by Tukey’s Honest Significant Difference (HSD) post hoc test for multiple comparisons. While ANOVA was primarily utilized to determine the significance of differences between treatment groups, the lack of significant variance among parallel replicates was used to confirm the reproducibility of the experimental measurements. Pearson’s correlation coefficient was calculated to examine the relationship between extract concentration and corresponding biological activities, as well as the association between antioxidant capacity and phenolic/flavonoid content. All statistical analyses were performed at a 95% confidence level, with *p* < 0.05 considered statistically significant.

## 3. Results

### 3.1. Antimicrobial Activity

#### 3.1.1. Disc Diffusion Results

Disk diffusion analyses were performed on 50, 100, and 150 microliter disks, and the following were calculated: 1.05, 2.10, and 3.15 mg of substance in ethanol, 2, 4, and 6 mg in methanol, 0.45, 0.90, and 1.35 mg in n-hexane, and 0.20, 0.40, and 0.60 mg in acetone, respectively. Detailed results for all reference strains are presented in Table 1.

In contrast, methanol and acetone extracts exhibited weak inhibition zones (7.0–9.0 mm) against specific strains such as *Bacillus cereus*, *Staphylococcus aureus*, and *Staphylococcus hominis*, particularly at higher concentrations (100–150 µL). The n-hexane extract demonstrated a broader spectrum of activity among the reference strains compared to other extracts, producing inhibition zones against *Bacillus subtilis* (8.0 mm), *B. cereus* (7.0–9.0 mm), *Listeria monocytogenes* (8.0–9.0 mm), and *S. aureus* (7.0–9.0 mm). The inhibition zone diameters of the positive controls are presented in Appendix A (Table A1); gentamicin exhibited inhibition zones ranging from 11 to 30 mm against all reference strains.

Detailed results of isolated microorganisms subjected to the same procedures are also presented in Table 2.

A clear susceptibility trend was observed regarding cell wall structures; Gram-positive bacteria (notably MRSA and *Enterococcus* species) were significantly more susceptible to the lipophilic extracts than Gram-negative bacteria. Statistical analysis via disk diffusion assays revealed highly significant differences in inhibition zone diameters among microorganisms (*p* < 2 × 10^−16^). While dose-dependent effects were significant for certain solvents like acetone (*p* = 0.0186) and methanol (*p* = 0.000418), Pearson correlation analyses showed weak and non-significant associations (e.g., ethanol: r = 0.105, *p* = 0.084; n-hexane: r = 0.020, *p* = 0.739). This statistical divergence is attributed to the high frequency of zero-value results (non-susceptible strains), which obscure linear dose–response correlations despite the overall significance of the observed activity. Finally, the lack of differences among parallel replicates confirms the high reliability and reproducibility of the experimental measurements.

#### 3.1.2. Minimum Inhibitory Concentration Results

The MIC and MBC analyses performed using ethanol, methanol, acetone, and *n*-hexane extracts of *G. aparine* revealed that the extracts were generally effective only at high concentrations. Detailed results are presented in Table 3.

The minimum inhibitory concentration (MIC) and minimum bactericidal concentration (MBC) values of *G. aparine* extracts across various microorganisms demonstrated significant variation depending on the solvent type and target strain (Table 3). Overall, the acetone extract exhibited the most potent antimicrobial activity among the tested solvents. Notably, it showed remarkable efficacy against *S. aureus* ATCC 25923, foodborne (FI) *E. durans*, and *E. faecium*, with remarkably low MIC values of 30 µg/mL. The lowest bactericidal effect (MBC) recorded for the acetone extract was observed against the *S. aureus* MRSA strain at a concentration of 1275 µg/mL.

The methanol extract displayed a broad spectrum of activity, particularly against *Pseudomonas fluorescens* P1, foodborne *E. faecium*, clinical isolate (CI) *S. aureus*, *Streptococcus mutans*, *Acinetobacter baumannii*, and the multidrug-resistant (MDR) *Providencia rustigianii* strain, all yielding an MIC of 710 µg/mL. The most effective MBC for the methanol extract was identified at 2303 µg/mL against *Salmonella infantis* (FI). Furthermore, the n-hexane extract demonstrated specific efficacy against foodborne *E. faecium* with an MIC of 165 µg/mL, while the ethanol extract showed limited activity, reaching its optimal performance against *Enterococcus faecalis* (MIC/MBC: 287 µg/mL).

Overall, the acetone extract emerged as the most potent, achieving the lowest MIC value of 30 µg/mL against *S. aureus* ATCC 25923, *E. durans* (FI), and *E. faecium* (FI). The n-hexane extract followed with specific efficacy against *E. faecium* (FI) at 165 µg/mL, while the ethanol extract reached its peak performance against *E. faecalis* (287 µg/mL). To distinguish between bacteriostatic and bactericidal activities, the MBC/MIC ratios were analyzed. According to the established criterion (MBC/MIC ≤ 4 for bactericidal and >4 for bacteriostatic), the ethanol extract exhibited a purely bactericidal profile against *E. faecalis* and *S. infantis* (ratio = 1). Conversely, the acetone extract displayed predominantly bacteriostatic effects against most strains, including *S. aureus* ATCC 25923 (ratio = 170), with the notable exception of its bactericidal activity against *S. aureus* MRSA (ratio = 4.0). Trends by solvent polarity indicate that moderately polar (acetone) and non-polar (n-hexane) extracts generally provided more effective inhibitory results than the highly polar (ethanol) extract. The high numerical values (e.g., >92,130 µg/mL) represent the maximum tested concentration limits for each specific solvent where total inhibition was not achieved, reflecting the limited susceptibility of certain strains to those particular extracts.

#### 3.1.3. Antibiofilm Activity Results

The microorganisms selected for biofilm assays were *E. faecalis* ATCC 29212, *S. infantis* (FI), *S. mutans* (CI), and multidrug-resistant (MDR) *S. aureus* MRSA. Prior to the assays, the glucose concentration and incubation period required for optimal biofilm formation were standardized for each strain. The optimal conditions were determined as follows: for *S. mutans*, 0.5% glucose after 24 h of incubation (0.117 ± 0.03); for *E. faecalis*, 1% glucose after 48 h (0.322 ± 0.06); for *S. infantis*, 0.5% glucose after 24 h (0.236 ± 0.07); and for *S. aureus* MRSA, 1.5% glucose after 24 h (0.085 ± 0.02).

Based on these parameters, biofilm assays were repeated with the addition of *G. aparine* extracts. The antibiofilm activities were evaluated at sub-inhibitory concentrations (MIC/2) to ensure that the observed reduction in biofilm formation was due to the inhibition of biofilm mechanisms rather than a general bactericidal effect. Detailed results are presented in Table 4.

**Figure 1:**
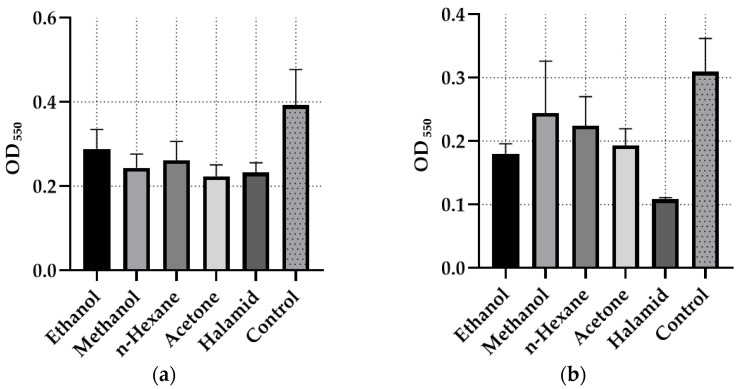
Absorbance readings (OD) of the extracts and Halamid tested for antibiofilm activity. (**a**) *Enterococcus faecalis* ATCC 29212, (**b**) *Salmonella infantis* (FI).

**Figure 2:**
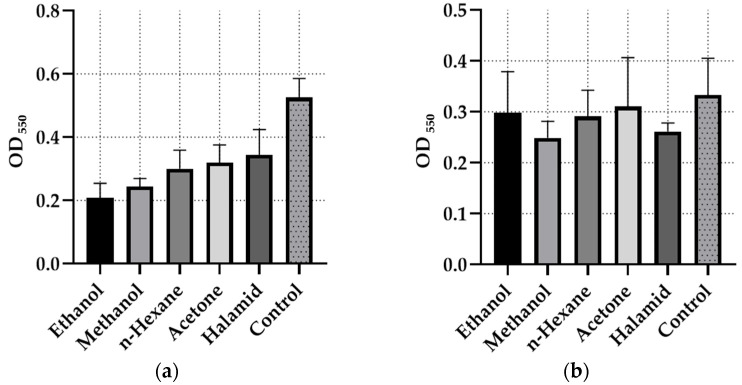
Absorbance readings (OD) of the extracts and Halamid tested for antibiofilm activity. (**a**) *Streptococcus mutans* (CI), (**b**) *Staphylococcus aureus* MRSA.

One-way ANOVA was performed to evaluate the biofilm inhibition effects of *G. aparine* extracts. Additionally, the absorbance values (OD_550_) are presented graphically in Figure 1 and Figure 2.

Among the tested isolates, *S. mutans* (CI) exhibited the highest biofilm inhibition, with the ethanol extract achieving 60.38 ± 0.04%. The acetone extract showed notable activity across multiple strains, reaching 43.06 ± 0.02% inhibition against *E. faecalis* ATCC 29212. Comparisons with the positive control, Halamid (1000 µg/mL), indicated that while Halamid inhibited *S. infantis* (FI) by 65.01 ± 0.01%, the ethanol extract showed higher inhibition (60.38 ± 0.04%) than Halamid (34.32 ± 0.06%) against *S. mutans*. The methicillin-resistant *S. aureus* MRSA strain demonstrated the lowest overall inhibition, with the methanol extract achieving 25.23 ± 0.03%, slightly exceeding Halamid’s effect (21.52 ± 0.06%). Overall, biofilm inhibition varied depending on the solvent and bacterial strain; for instance, *S. infantis* was strongly inhibited by ethanol (41.97 ± 0.01%) but minimally affected by methanol (21.08 ± 0.07%).

The Shapiro–Wilk test results indicated that the data were normally distributed for all isolates (*p* > 0.05), and the Levene test confirmed the homogeneity of variances (*p* > 0.05).

In the *S. mutans* clinical isolate, no statistically significant difference was observed in antibiofilm activity among the solvents (*p* = 0.0675), although the result was close to the threshold of significance. For the *S. infantis* isolate, a significant difference was detected among solvents (*p* = 0.0298). According to Tukey’s post hoc test, halamid exhibited higher inhibition activity than methanol and, to a lesser extent, n-hexane extracts (*p* = 0.0236 and *p* = 0.0559, respectively). In contrast, no significant differences were found among solvents for *S. aureus* MRSA and *E. faecalis* ATCC 29212 strains (*p* > 0.05).

Two-way ANOVA results revealed that the bacterial species factor had a highly significant effect on antibiofilm activity (F(3,40) = 24.476, *p* = 3.71 × 10^−9^), whereas the solvent type factor was not statistically significant (*p* = 0.173). The bacteria–solvent interaction showed a borderline significance (*p* = 0.059).

Among the tested extracts, the ethanol extract emerged as the most effective antibiofilm agent, particularly against *Streptococcus mutans* (CI) with a maximum inhibition of 60.38%; notably, this high antibiofilm activity was achieved at sub-inhibitory concentrations (MIC/2) where direct antimicrobial growth inhibition was absent, suggesting that the effect is independent of the extract’s bactericidal potency.

#### 3.1.4. Anti-Quorum Sensing Activity (AQS) Results

**Figure 3:**
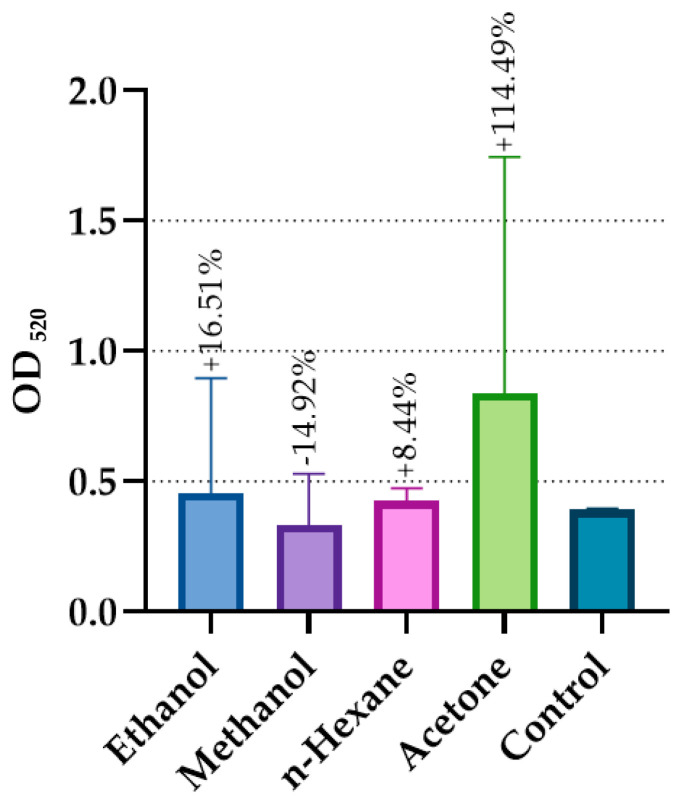
Inhibitory effects of different solvent extracts on pyocyanin production in *Pseudomonas aeruginosa* PA01.

The anti-quorum sensing activity of *G. aparine* extracts against *Pseudomonas aeruginosa* PA01 was evaluated, and the corresponding OD values are presented in Figure 3.

The anti-quorum sensing activity of *G. aparine* extracts against *P. aeruginosa* PA01 was evaluated by measuring the inhibition of pyocyanin production. The ethanol extract demonstrated the most potent AQS activity with an inhibition rate of 46.16 ± 9.28%. Moderate inhibitory effects were observed for acetone (19.18 ± 2.81%) and methanol (14.92 ± 5.23%) extracts, while the n-hexane extract resulted in a slight increase (−8.44 ± 12.79%) in pigment production. To ensure that the observed reduction in pyocyanin was independent of growth suppression, all values were normalized to the cell density (OD_520_/OD_600_), and no significant growth inhibition was observed at the tested concentrations. The percentage of inhibition was calculated using the formula:

Inhibition % = [1 − (ODsample/ODcontrol)] × 100

Statistical analysis via ANOVA confirmed that the extraction solvent significantly influenced AQS activity (*p* = 0.00517). Although the overall ANOVA was significant, the lack of significance in certain Tukey post hoc pairwise comparisons (*p* > 0.05) is attributed to the high standard deviation in the n-hexane and ethanol groups. Biologically, the 46% inhibition achieved by the ethanol extract is highly relevant, as it represents a nearly twofold reduction in a key virulence factor without exerting lethal selective pressure on the bacteria.

### 3.2. Antioxidant Activity Results

#### 3.2.1. DPPH Radical Scavenging Activity

**Figure 4:**
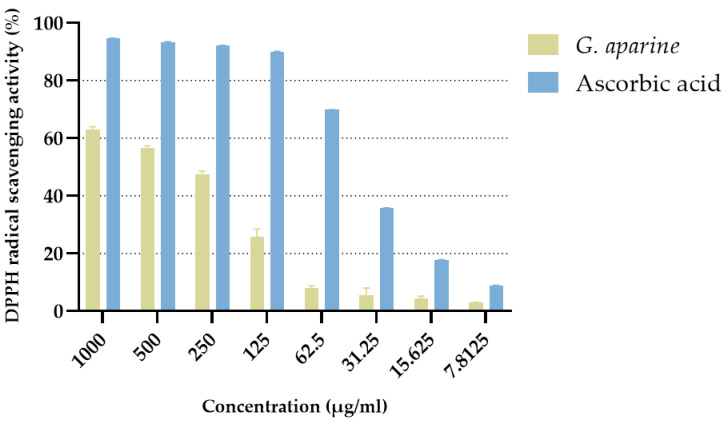
DPPH radical scavenging activity (%) of *Galium aparine* ethanol extract and ascorbic acid.

The DPPH radical scavenging activity was evaluated for both the standard antioxidant ascorbic acid and ethanol extract of *Galium aparine* at different concentrations. Ascorbic acid exhibited high radical scavenging capacity even at low concentrations, starting at 11.8 ± 0.0431% at 0.00781 mg/mL and increasing rapidly with concentration to reach 94.7 ± 0.0025% at 1 mg/mL. A detailed summary of the results is provided in Figure 4.

The extract showed lower antioxidant activity compared to ascorbic acid. At 0.00781 mg/mL, the radical scavenging rate was 2.86 ± 0.265%, which increased linearly with concentration and reached 62.9 ± 0.990% at 1 mg/mL. Notably, at medium and high concentrations, the extract’s activity increased significantly, demonstrating a clear dose-dependent effect.

EC_50_ values calculated using the four-parameter log-logistic (4PL) model were 0.1592 ± 0.0039 mg/mL for the extract and 0.04004 ± 0.00054 mg/mL for ascorbic acid, indicating that the extract achieved half-maximal effect at a higher concentration than ascorbic acid. ANOVA analyses revealed that extract and ascorbic acid, and also concentration, had statistically significant effects on DPPH radical scavenging activity (*p* < 2.0 × 10^−16^). One-way ANOVA on the extract data confirmed that concentration significantly affected its activity (*p* < 2.0 × 10^−16^), while no significant differences were observed between parallel replicates (*p* = 0.9986). Pearson correlation analysis revealed a strong and positive relationship between extract concentration and DPPH scavenging activity (r = 0.8823, 95%, *p* < 2.2 × 10^−16^), confirming that antioxidant activity increased with increasing concentration.

#### 3.2.2. CUPRAC

The absorbance values of the ascorbic acid standard solutions were measured at 450 nm, and a consistent increase in absorbance was observed with increasing concentrations. Linear regression analysis yielded the standard calibration equation for ascorbic acid as y = 4.949x + 0.209 (R^2^ = 0.984, *p* = 1.08 × 10^−5^). This high coefficient determination indicates a strong linear relationship, confirming the reliability of the CUPRAC method. Ethanol extract of *G. aparine* was analyzed individually, and the ascorbic acid equivalents (AAE) were calculated using the standard curve equation. The AAE value for the extract is 0.763 ± 0.010 mg ascorbic acid/mL.

#### 3.2.3. ABTS

The ethanol extract of *G. aparine* at 1 mg/mL exhibited a strong inhibitory effect, with three independent measurements yielding OD values of 0.084, 0.081, and 0.087. This corresponded to a mean inhibition of 88.10 ± 0.25% compared to the control (OD = 0.706 ± 0.004). As a positive control, ascorbic acid (1 mg/mL) was included, resulting in 87.3 ± 0.21% inhibition.

#### 3.2.4. FRAP

The reducing power of *G. aparine* ethanol extract was evaluated using the FRAP assay and compared to the standard antioxidant, ascorbic acid. The ascorbic acid standard curve showed a strong linear relationship between concentration and absorbance, with the regression equation y = 0.014 × x + 0.145 (R^2^ = 0.9998), indicating excellent linearity and reproducibility of the assay. The ethanol extract exhibited absorbance values of three independent measurements. When these values were converted to AAE using the standard curve, the extract showed AAE of 24.36 ± 4.16 mg/L.

### 3.3. Biochemical Analysis Results of Galium Aparine Extract

The GC-MS analysis revealed that the ethanol extract of *G. aparine* included a few different chemical components. Twenty compounds were found in all. Table 5 offers comprehensive details on the chemicals that have been found.

In this study, the chemical composition of the ethanolic extract of *G. aparine* was analyzed using GC–MS. The analysis identified a total of 22 compounds, including two unidentified ones, most of which were fatty acids and their esters (Table 5). This finding indicates that the ethanolic extract of *G. aparine* possesses a chemically rich profile dominated by lipophilic compounds.

According to the results, the most abundant constituents of the extract were palmitic acid (31.25%), linolenic acid (20.64%), and linoleic acid (13.28%). Together, these three compounds accounted for approximately 65.17% of the total composition, playing a dominant role in defining the chemical profile of the extract. The high proportion of unsaturated fatty acids suggests that the extract may serve as a potential source of antioxidant, anti-inflammatory, and antimicrobial agents.

In addition, notable amounts of terpenoid compounds such as neophytadiene (6.15%), phytol palmitate (5.01%), and phytol (1.68%) were detected in the extract. Minor components including ethyl palmitate (1.78%), stearic acid (1.63%), myristic acid (0.91%), and alloaromadendrene oxide (0.54%) also contributed to the overall chemical composition.

Overall, the GC–MS analysis demonstrated that the ethanolic extract of *G. aparine* is predominantly composed of saturated and unsaturated long-chain fatty acids and terpenoid secondary metabolites. These compounds are likely to play a key role in ex-plaining the extract’s biological activities. Therefore, the ethanolic extract of *G. aparine* bioactive compounds was accessed compared to earlier studies that relied on similar extraction approaches. Moreover, the antimicrobial potential of *G. aparine* was evaluated on an unprecedented scale, encompassing 45 strains, including standard, foodborne, clinical, and multidrug-resistant isolates—thus offering one of the most extensive perspectives in the literature.

Although all obtained extracts (methanol, acetone, n-hexane, and ethanol) were subjected to GC-MS analysis under identical conditions, a well-defined chromatographic profile was exclusively achieved for the ethanol extract. In the cases of methanol, acetone, and n-hexane extracts, the concentration of secondary metabolites remained below the analytical threshold for robust mass spectrometric detection. The absence of significant peaks in these solvents can be attributed to the specific solubility limits and the low signal-to-noise ratio encountered during the scanning process, which prevented reliable qualitative and quantitative identification of individual compounds. Consequently, to ensure data integrity and avoid speculative characterization, only the chemically significant results belonging to the ethanol extract are presented and discussed in this study.

## 4. Discussion

This study represents, to our knowledge, one of the most comprehensive investigations to date into the biological activities and biochemical composition of *G. aparine*, advancing beyond the scope of previous reports. While earlier studies have provided valuable but limited insights, our findings not only confirm existing knowledge but also expand it significantly. By employing a distinct solvent system (methanol, acetone, and n-hexane), a broader spectrum of bioactive compounds was accessed compared to earlier studies that relied on similar extraction approaches. Moreover, the antimicrobial potential of *G. aparine* was evaluated on an extensive scale, encompassing 45 strains, including standard, foodborne, clinical, and multidrug-resistant isolates—thus offering one of the broadest perspectives in the literature.

The results of the disk diffusion and MIC assays highlight the significant role of solvent polarity in extracting bioactive metabolites. While the ethanol extract of *G. aparine* exhibited no activity against standard strains in disk diffusion, the methanol, acetone, and n-hexane extracts showed clear antimicrobial effects, with the strongest inhibition zone observed for the n-hexane extract (Table 1). This suggests that non-polar constituents may possess higher diffusivity in agar-based assays. When compared with previous studies, Ilyina et al. [59] reported higher inhibition zones, likely due to their use of solvent mixtures at varying ratios, whereas the present study utilized single-solvent extractions. Similarly, Goryacha et al. [60] reported stronger inhibition for common strains. This discrepancy can reasonably be explained by variations in the geographic origin of plant material, which is known to influence secondary metabolite composition. Despite these differences, the findings of Ilyina et al. [59], Goryacha et al. [60], and the present study consistently support the conclusion that *G. aparine* exhibits noteworthy antimicrobial activity.

In the MIC evaluation, the highest activity was observed against *S. aureus* ATCC 25923, *E. durans* (FI), and *E. faecium* (FI) strains, with an MIC value of 30 µg/mL. In contrast, Beirami et al. [61] reported the strongest activity against *S. aureus* with a much higher MIC value of 50,000 µg/mL. The considerable difference is likely due to variations in geographical origin and solvents used. The dominant compounds identified—palmitic acid (31.25%), linolenic acid (20.64%), and linoleic acid (13.28%)—may contribute to these effects. These long-chain fatty acids are known to disrupt the cytoplasmic membrane, a mechanism particularly effective against Gram-positive bacteria, which lack the protective outer lipopolysaccharide layer. This potentially explains the significantly lower MIC values observed against *Staphylococcus aureus* and *Enterococcus* strains. Notably, the strong inhibitory effect against *S. aureus* MRSA is of great significance, as it is a prevalent multidrug-resistant pathogen [62] responsible for severe systemic infections [63]. Given that individuals infected with MRSA have a 64% higher risk of mortality [64], *G. aparine* highlights a potential relevance in developing adjunct natural therapies targeting antibiotic-resistant pathogens.

This study provides a pioneering report on the antibiofilm and AQS activities of *G. aparine*. Biofilms form a protective shield for bacteria and play a central role in chronic infections and antibiotic resistance [65]. Our analyses demonstrated the capacity of *G. aparine* extracts to inhibit biofilm formation, with the ethanol extract achieving the highest inhibition against *S. mutans* (CI), markedly surpassing the positive control, Halamid. Two-Way ANOVA results confirmed that the bacterial species factor had a highly significant effect (*p* = 3.71 × 10^−9^). Given its significant biofilm inhibition, these results suggest that *G. aparine* holds potential as a natural component in oral hygiene applications or topical formulations as an adjunct therapy.

The inhibition of the quorum sensing (QS) system, which enables bacterial coordination, is vital for preventing biofilm formation [66,67]. In this study, the ethanol extract exhibited the highest anti-quorum sensing (AQS) effect (–46.16 ± 9.28%) against *P. aeruginosa* PA01. It should be clarified that the negative sign (−) indicates a percentage reduction in pyocyanin production relative to the control, signifying strong inhibition. Terpenoids such as neophytadiene (6.15%) and phytol (1.68%) likely act synergistically with the fatty acid profile to facilitate this. These lipophilic molecules can potentially interfere with bacterial cell-to-cell communication signaling pathways and the stability of the extracellular polymeric substance (EPS) matrix.

Antioxidant potential was assessed using multiple assays (DPPH, ABTS, CUPRAC, FRAP), proving that the extract functions as both a free radical scavenger and a reducing agent. In the ABTS assay, the extract showed an inhibition (88.10 ± 0.25%) nearly equivalent to ascorbic acid. DPPH results also supported a dose-dependent effect (r = 0.8823). Neelam and Khan [68] and Ozmatara [69] also reported significant antioxidant activity for *G. aparine*, and while numerical differences exist due to solvent choice or standards used (e.g., Trolox vs. Ascorbic Acid [70]), these studies mutually support the plant’s efficacy.

The GC-MS analysis of ethanol extract revealed a diverse range of chemical classes, including fatty acids, hydrocarbons, terpenoids, and esters (Table 5). Dominant constituents like palmitic, linolenic, and linoleic acids are known for antimicrobial and antioxidant properties [71,72,73,74]. Compounds such as neophytadiene and phytyl palmitate further support this bioactivity [75]. The presence of phenolic compounds, though not fully characterized here, likely plays a major role in the observed antioxidant and reducing power mechanisms observed in the CUPRAC and FRAP assays.

While these findings are promising, several limitations must be addressed. The current experiments are purely in vitro, and the lack of cytotoxicity testing is a critical gap that must be filled before suggesting therapeutic use. Furthermore, the mechanistic validation of AQS inhibition is limited as no gene expression analysis was performed to confirm the downregulation of QS-related genes. It is also important to note that GC-MS is restricted to volatile and semi-volatile compounds, potentially overlooking polar metabolites. Finally, because only the ethanol extract was tested for antioxidant activity, the interpretation may be biased toward that specific solvent’s recovery capacity.

## 5. Conclusions

This study aimed to investigate the biochemical composition and various biological activities of *G. aparine* extracts through a comprehensive multi-solvent approach. Our findings demonstrate that the plant possesses significant antimicrobial potency, particularly against multidrug-resistant pathogens such as MRSA, and exhibits a multi-mechanistic antioxidant capacity. Notably, this research provides the first evidence of the plant’s anti-quorum sensing and antibiofilm activities, with the ethanol extract showing remarkable efficacy against *S. mutans*. These results provide scientific support for certain aspects of the traditional medicinal use of *G. aparine*, particularly regarding its potential role in managing infections.

However, it is important to acknowledge that the present findings are based on in vitro assays. Further in vivo studies and comprehensive toxicity evaluations are required to confirm the therapeutic safety and clinical efficacy of these extracts. The scientific contribution of this work lies in identifying *G. aparine* as a promising source of lipophilic bioactive compounds, yet the exact molecular mechanisms underlying its quorum sensing inhibition remain to be fully elucidated.

Future research should focus on the bioactivity-guided isolation and characterization of specific active compounds to determine their individual contributions. Additionally, detailed mechanistic studies on quorum sensing pathways and the development of specialized topical or oral hygiene formulations will be essential to translate these laboratory findings into practical therapeutic applications.

## Figures and Tables

**Table 1:** Disk diffusion results of *Galium aparine* extracts against reference strains (mm).

Microorganisms	Ethanol ^a^			Methanol ^a^			Acetone ^a^			*n*-Hexane ^a^		
	50	100	150	50	100	150	50	100	150	50	100	150
*Acinetobacter baumannii* CECT 9111	R	R	R	R	R	R	R	R	R	R	R	R
*Bacillus subtilis *DSM 1971	R	R	R	R	R	R	R	R	R	8.00 ± 0.00	8.33 ± 0.58	8.33 ± 0.58
*Bacillus cereus *RSKK 863	R	R	R	R	R	R	7.33 ± 0.58	8.00 ± 0.00	8.00 ± 0.00	7.00 ± 0.00	7.66 ± 0.58	9.00 ± 0.00
*Enterobacter aerogenes *ATCC 13048	R	R	R	7.00 ± 0.00	7.00 ± 0.00	7.00 ± 0.00	7.00 ± 0.00	7.00 ± 0.00	7.00 ± 0.00	R	R	R
*Enterococcus faecalis *ATCC 29212	R	R	R	R	R	R	R	R	R	R	R	R
*Escherichia coli *ATCC 25922	R	R	R	R	R	R	R	R	R	R	R	R
*Listeria monocytogenes *ATCC 7644	R	R	R	R	R	R	R	R	R	8.00 ± 0.00	8.66 ± 0.58	0.00 ± 0.00
*Pseudomonas aeruginosa *DSM 50071	R	R	R	R	R	R	7.00 ± 0.00	7.00 ± 0.00	7.00 ± 0.00	7.00 ± 0.00	8.00 ± 0.00	9.00 ± 0.00
*Pseudomonas fluorescens *P1	R	R	R	7.00 ± 0.00	7.00 ± 0.00	7.00 ± 0.00	R	R	R	R	R	R
*Salmonella enteritidis *ATCC 13076	R	R	R	R	R	R	R	R	R	R	R	R
*Salmonella typhimurium *SL 1344	R	R	R	7.00 ± 0.00	7.00 ± 0.00	7.00 ± 0.00	R	R	R	R	R	R
*Staphylococcus aureus *ATCC 25923	R	R	R	8.00 ± 0.00	8.00 ± 0.00	8.00 ± 0.00	8.00 ± 0.00	8.00 ± 0.00	8.00 ± 0.00	7.00 ± 0.00	7.66 ± 0.58	9.00 ± 0.00
*Staphylococcus epidermidis *DSM 20044	R	R	R	R	R	7.66 ± 0.58	8.00 ± 0.00	8.00 ± 0.00	8.00 ± 0.00	R	R	R
*Staphylococcus hominis *ATCC 27844	R	R	R	R	8.00 ± 0.00	8.00 ± 0.00	R	7.00 ± 0.00	8.00 ± 0.00	7.33± 0.58	8.00 ± 0.00	9.00 ± 0.00
*Staphylococcus warneri *ATCC 27836	R	R	R	R	R	R	R	R	R	R	R	R
*Shigella flexneri *RSKK 184	R	R	R	R	R	R	8.00 ± 0.00	R	R	R	R	R

^a^ The discs were loaded with 50, 100 and 150 µL of extracts. Mean ± standard deviation is used to report values (n = 3). R: Resistant. Overall, the antimicrobial effects of the *Galium aparine* extracts were observed to be limited. Regarding the reference strains (Table 1), the ethanol extract showed no antimicrobial activity at any tested concentration (0.00 mm).

**Table 2:** Disk diffusion results of *Galium aparine* extracts against isolate strains (mm).

Microorganisms	Ethanol ^a^			Methanol ^a^			Acetone ^a^			*n*-Hexane ^a^		
	50	100	150	50	100	150	50	100	150	50	100	150
*Achromobacter* sp. (MDR)	R	7.00 ± 0.00	8.00 ± 0.00	7.00 ± 0.00	7.00 ± 0.00	7.00 ± 0.00	8.00 ± 0.00	8.00 ± 0.00	8.00 ± 0.00	R	R	R
*Acinetobacter baumannii *(CI)	R	R	R	8.00 ± 0.00	8.00 ± 0.00	8.00 ± 0.00	R	R	R	R	R	R
*Acinetobacter baumannii *(MDR)	R	R	R	7.00 ± 0.00	7.00 ± 0.00	7.00 ± 0.00	R	R	R	R	R	R
*Enterobacter aerogenes *(MDR)	R	R	R	R	R	R	9.00 ± 0.00	9.00 ± 0.00	9.00 ± 0.00	R	R	R
*Enterococcus durans *(FI)	R	R	R	R	R	R	8.00 ± 0.00	8.00 ± 0.00	8.00 ± 0.00	R	R	R
*Enterococcus faecalis *(CI)	R	R	R	R	R	R	R	R	R	R	R	R
*Enterococcus faecium *(FI)	R	R	R	R	R	7.00 ± 0.00	10.00 ± 0.00	10.33 ± 0.58	11.00 ± 0.00	7.00 ± 0.00	7.00 ± 0.00	7.00 ± 0.00
*Escherichia coli *(FI)	R	R	R	R	R	R	R	R	R	R	R	R
*Escherichia coli *(MDR)	R	R	R	7.00 ± 0.00	7.00 ± 0.00	7.00 ± 0.00	R	R	R	R	R	R
*Klebsiella pneumoniae *(CI)	R	R	R	R	R	R	R	R	R	R	R	R
*Klebsiella pneumoniae *(FI)	R	R	R	R	R	R	R	R	R	R	R	R
*Klebsiella pneumoniae *(MDR)	R	R	R	R	R	R	7.00 ± 0.00	7.00 ± 0.00	7.00 ± 0.00	R	8.00 ± 0.00	9.00 ± 0.00
*Listeria innocua *(FI)	R	R	R	R	R	R	7.00 ± 0.00	R	R	7.00 ± 0.00	7.00 ± 0.00	7.00 ± 0.00
*Proteus vulgaris *(MDR)	R	R	R	R	R	R	R	R	R	7.00 ± 0.00	7.00 ± 0.00	7.00 ± 0.00
*Providencia rustigianii *(MDR)	R	R	R	8.33 ± 0.58	8.33 ± 0.58	9.00 ± 0.00	8.00 ± 0.00	8.00 ± 0.00	8.00 ± 0.00	11.33 ± 0.58	13.33 ± 0.58	15.33 ± 0.58
*Salmonella infantis *(FI)	R	R	R	R	R	R	R	R	R	R	R	R
*Salmonella kentucky *(FI)	R	R	R	7.00 ± 0.00	7.00 ± 0.00	7.00 ± 0.00	8.00 ± 0.00	R	R	R	R	R
*Serratia odorifera *(MDR)	R	R	R	7.00 ± 0.00	7.00 ± 0.00	7.00 ± 0.00	R	R	R	R	R	R
*Shigella boydi *(CI)	R	R	R	R	R	R	R	R	R	R	R	R
*Shigella flexneri *(CI)	R	R	R	7.00 ± 0.00	7.00 ± 0.00	7.00 ± 0.00	R	R	R	R	R	R
*Staphylococcus aureus *(CI)	R	R	R	7.00 ± 0.00	7.00 ± 0.00	7.00 ± 0.00	7.33 ± 0.58	10.00 ± 0.00	10.00 ± 0.00	11.00 ± 0.00	10.33 ± 0.58	9.00 ± 0.00
*Staphylococcus aureus *2 (CI)	R	R	R	R	R	R	R	R	R	R	R	R
*Staphylococcus aureus *MRSA	9.00 ± 0.00	10.00 ± 0.00	11.00 ± 0.00	10.00 ± 0.00	12.00 ± 0.00	12.00 ± 0.00	8.00 ± 0.00	11.00 ± 0.00	11.00 ± 0.00	15.00 ± 0.00	18.00 ± 0.00	19.00 ± 0.00
*Staphylococcus aureus *MRSA 2	R	R	R	R	8.00 ± 0.00	8.00 ± 0.00	8.00 ± 0.00	8.00 ± 0.00	8.00 ± 0.00	11.33 ± 0.58	13.33 ± 0.58	15.33 ± 0.58
*Staphylococcus haemolyticus *(CI)	R	R	R	R	R	R	R	R	R	R	R	R
*Staphylococcus hominis *(CI)	R	R	R	R	R	R	R	R	R	R	R	R
*Staphylococcus lugdunensis *(CI)	R	R	R	R	R	R	R	R	R	R	R	R
*Streptococcus mutans *(CI)	R	7.00 ± 0.00	8.00 ± 0.00	9.00 ± 0.00	9.33 ± 0.58	10.00 ± 0.00	7.00 ± 0.00	8.00 ± 0.00	0.00 ± 0.00	8.00 ± 0.00	9.33 ± 0.58	10.00 ± 0.00
*Streptococcus pneumoniae *(MDR)	R	R	R	7.00 ± 0.00	7.00 ± 0.00	7.00 ± 0.00	R	R	R	R	R	R

^a^ The discs were loaded with 50, 100 and 150 µL of extracts. Mean ± standard deviation is used to report values (n = 3). CI: Clinical isolate; FI: foodborne; MDR: Multi drug resistance, MRSA: Methicillin resistance *Staphylococcus aureus*. R: Resistant. The antimicrobial activity of *G. aparine* extracts against isolate strains was characterized as selective and generally weak (<10 mm) to moderate (10–15 mm), with specific instances of strong activity (>15 mm) observed for the n-hexane extract. Unlike its performance against reference strains, the ethanol extract exhibited localized activity against specific isolates, notably *Achromobacter* sp. (MDR) (7.0–8.0 mm), *S. aureus* MRSA (9.0–11.0 mm), and *Streptococcus mutans* (CI) (7.0–8.0 mm). The methanol extract produced inhibition zones ranging from 7.0 to 12.0 mm, showing notable activity against *Achromobacter* sp. (MDR), *Providencia rustigianii* (MDR), and *S. aureus* MRSA. The acetone extract demonstrated its most pronounced effects against *Enterococcus faecium* (FI) (10.0–11.0 mm) and *Enterobacter aerogenes* (MDR) (9.0 mm), while remaining inactive against *Escherichia coli* (MDR). The n-hexane extract displayed the highest antimicrobial potential among all solvents, particularly against multi-drug resistant (MDR) isolates. The most significant inhibition zones—categorized as strong activity—were recorded against *S. aureus* MRSA (15.0–19.0 mm), *P. rustigianii* (MDR) (11.0–15.0 mm), and *S. aureus* MRSA 2 (11.0–15.0 mm). In contrast, no inhibition was detected against *Klebsiella pneumoniae* or *Escherichia coli* (FI) strains. The positive control, gentamicin, maintained strong activity (7.0–28 mm) across all tested isolates (Appendix A, Table A1).

**Table 3:** MIC and MBC results of *Galium aparine* extracts (µg/mL).

Microorganisms	Ethanol ^a^		Methanol ^a^		Acetone ^a^		n-Hexane ^a^	
	MIC	MBC	MIC	MBC	MIC	MBC	MIC	MBC
*Bacillus subtilis* DSM 1971	-	-	-	-	-	-	>21,130	>21,130
*Listeria monocytogenes* ATCC 7644	-	-	-	-	-	-	660	10,565
*Enterobacter aerogenes* ATCC 13048	-	-	>92,130	>92,130	>5100	>5100	-	-
*Enterococcus faecalis* ATCC 29212	287	287	710	>710	150	>150	660	10565
*Pseudomonas aeruginosa DSM 50071*	-	-	-	-	>5100	>5100	>21,130	>21,130
*Pseudomonas fluorescens* P1	-	-	710	>92,130	-	-	-	-
*Salmonella typhimurium* SL 1344	-	-	92,130	>92,130	-	-	-	-
*Staphylococcus aureus* ATCC 25923	-	-	1430	46,060	30	5100	>21,130	>21,130
*Staphylococcus epidermidis* DSM 20044	-	-	23,030	92,130	2550	5100	-	-
*Staphylococcus hominis* ATCC 27844	-	-	11,510	>92,130	1275	5100	>21,130	>21,130
*Bacillus cereus RSKK 863*	-	-	-	-	1275	5100	>21,130	>21,130
*Shigella flexneri RSKK 184*	-	-	-	-	5100	>5100	-	-
*Enterococcus durans* (FI)	-	-	-	-	30	>5100	-	-
*Enterococcus faecium* (FI)	-	-	710	92,130	30	5100	165	>21,130
*Listeria innocua* (FI)	-	-	-	-	1275	5100	2641	21,130
*Salmonella kentucky* (FI)	-	-	>92,130	>92,130	>5100	>5100	-	-
*Salmonella infantis* (FI)	575	575	2303	2303	630	5100	2641	2641
*Staphylococcus aureus* (CI)	-	-	710	23,030	315	2550	-	-
*Streptococcus mutans* (CI)	575	>575	710	46,060	630	5100	2641	21,130
*Staphylococcus haemolyticus* (CI)	-	-	-	-	630	5100	-	-
*Acinetobacter baumannii* (CI)	-	-	710	>92,130	-	-	-	-
*Shigella flexneri* (CI)	-	-	23,030	-	-	-	-	-
*Escherichia coli* (MDR)	-	-	46,060	-	-	-	-	-
*Klebsiella pneumoniae* (MDR)	-	-	-	-	5100	5100	>21,130	>21,130
*Enterobacter aerogenes* (MDR)	-	-	-	-	>5100	>5100	-	-
*Acinetobacter baumannii* (MDR)	-	-	92,130	-	-	-	-	-
*Serratia odorifera* (MDR)	-	-	710	>92,130	-	-	-	-
*Streptococcus pneumoniae* (MDR)	-	-	>92,130	>92,130	-	-	-	-
*Proteus vulgaris* (MDR)	-	-	-	-	-	-	10,565	10,565
*Staphylococcus aureus* MRSA	9200	>9200	710	23,030	315	1275	1320	5282
*Staphylococcus aureus* MRSA 2	-	-	1430	23,030	630	5100	660	10,565
*Providencia rustigianii* (MDR)	-	-	710	23,030	315	2550	660	5282
*Achromobacter* sp. (MDR)	>36,800	>36,800	2870	46,060	157	>5100	-	-

^a^ The solvent was evaporated, and the extracts were prepared using DMSO. A dash (-) indicates that the test was not performed; CI: Clinical isolate; FI: foodborne; MDR: Multi drug resistance, MRSA: Methicillin resistance *Staphylococcus aureus*.

**Table 4:** Biofilm inhibition results of *Galium aparine* extracts and Halamid (%).

Microorganisms	Ethanol ^a^	Methanol ^a^	Acetone ^a^	*n*-hexane ^a^	Halamid
	MIC/2	MIC/2	MIC/2	MIC/2	1000 µg/mL
*Enterococcus faecalis* ATCC 29212	26.43 ± 0.04	37.97 ± 0.03	43.06 ± 0.02	33.47 ± 0.04	40.85 ± 0.02
*Salmonella infantis *(FI)	41.97 ± 0.01	21.08 ± 0.07	37.55 ± 0.02	27.54 ± 0.04	65.01 ± 0.01
*Streptococcus mutans *(CI)	60.38 ± 0.04	53.53 ± 0.02	39.08 ± 0.04	42.82 ± 0.05	34.32 ± 0.06
*Staphylococcus aureus *MRSA	10.31 ± 0.07	25.23 ± 0.03	6.71 ± 0.08	12.61 ± 0.04	21.52 ± 0.06

^a^ The solvent was evaporated, and the extracts were prepared using DMSO. Negative values indicate an increase in biofilm formation at that concentration; CI: Clinical isolate; FI: foodborne. Mean ± standard deviation is used to report values (n = 3).

**Table 5:** GC-MS analysis results of *Galium aparine* Ethanol extract.

Compound Name	Retention Time	Formula	Molecular Weight	Area (%)	Known Activity
(1S,6R)-3,7,7-trimethylbicyclo [4.1.0]hept-3-en-5-one	21.476	C_10_H_14_O	150.22	0.80	-
Octadec-9-ene	23.812	C_18_H_36_	252.5	1.06	-
Curcumene	26.648	C_15_H_22_	202.33	0.51	Antimicrobial [43]
p-Cymene	29.639	C_10_H_14_	134.22	1.06	Antioxidant and Antimicrobial [44,45]
1-Hexadecene	29.866	C_16_H_32_	224.42	0.75	Antioxidant and Antimicrobial [46]
Myristic Acid	34.998	C_14_H_28_O_2_	228.37	0.91	Antifungal, Antiviral, Anticancer and Antiparasitic [47]
1-Octadecene	35.313	C_10_H_36_	252.5	0.77	-
Neophytadiene	36.503	C_20_H_38_	278.5	6.15	Neuropharmacological effect [48]
6,10,14-Trimethyl-2-pentadecanone	36.700	C_18_H_36_O	268.5	1.89	-
Unknown	37.452	-	-	2.29	-
Tricyclo[4.3.1.0(2,5)]decane	39.388	C_10_H_16_	136.23	2.12	-
Palmitic Acid	40.225	C_16_H_32_O_2_	256.42	31.25	Antitumor and Antiviral [49,50]
Ethyl palmitate	40.347	C_18_H_36_O_2_	284.5	1.78	Anti-inflammatory and Acaricidal [51,52]
Alloaromadendrene oxide-(1)	41.252	C_15_H_24_O	220.35	0.54	-
Phytol	43.099	C_20_H_40_O	296.5	1.68	Antiradical and Antimicrobial [53]
Linoleic Acid	44.026	C_18_H_32_O_2_	280.4	13.28	Antioxidant and Antimalarial [54,55]
Linolenic Acid	44.205	C_18_H_30_O_2_	278.4	20.64	Antibacterial [56]
Stearic Acid	44.586	C_18_H_36_O_2_	284.5	1.63	Antibacterial [57]
Octadecane	56.727	C_18_H_38_	254.5	0.45	-
Nonadecane	61.264	C_19_H_40_	268.5	0.85	Antioxidant and Antidiabetic [58]
Unknown	63.326	-	-	4.60	-
Phytyl palmitate	79.122	C_36_H_70_O_2_	534.9	5.01	-

A dash (-) indicates the absence of the respective molecule; C: Carbon; H: Hydrogen; O: Oxygen; Cl: Chlorine; N: Nitrogen. Identification Method: RT and MS Library (Wiley-Nist).

## Data Availability

The original contributions presented in this study are included in the article. Further inquiries can be directed to the corresponding author.

## Abbreviations

The following abbreviations are used in this manuscript:

MIC	Minimum Inhibitory Concentration
MBC	Minimum Bactericidal Concentration
ABTS	2,2′-Azino-bis(3-ethylbenzothiazoline-6-sulfonic acid)
DPPH	2,2-Diphenyl-1-picrylhydrazyl
FRAP	Ferric Reducing Antioxidant Power
CUPRAC	Cupric Reducing Antioxidant Capacity
GC–MS	Gas Chromatography–Mass Spectrometry
AQS	Anti-Quorum Sensing
EtOH	Ethanol
MeOH	Methanol
AMR	Antimicrobial Resistance
MRSA	Methicillin Resistance *Staphylococcus aureus*
MDR	Multi Drug Resistance
XDR	Extensively Drug-Resistant
FI	Foodborne
CI	Clinical Isolate
ROS	Reactive Oxygen Species
DMSO	Dimethyl Sulfoxide
MHA	Mueller Hinton Agar
MHB	Mueller–Hinton Broth
WHO	World Health Organization
AAE	Ascorbic Acid Equivalents

## Appendix A. Antibiotic Results

The results of antibiotic discs are presented in Table A1.

**Table A1:** Disk diffusion results of Gentamicin and Clindamycin against strains (mm).

Microorganisms	Gentamicin	Clindamycin
	10 µg	10 µg
*Achromobacter* sp. (MDR)	9	R
*Acinetobacter baumannii* (CI)	15	R
*Acinetobacter baumannii* (MDR)	R	R
*Acinetobacter baumannii* CECT 9111	13	R
*Bacillus cereus* RSKK 863	24	26
*Bacillus subtilis* DSM 1971	30	34
*Enterobacter aerogenes* (MDR)	16	R
*Enterobacter aerogenes* ATCC 13048	24	R
*Enterococcus durans* (FI)	11	30
*Enterococcus faecalis* (CI)	24	9
*Enterococcus faecalis* ATCC 29212	12	R
*Enterococcus faecium* (FI)	28	30
*Escherichia coli* (FI)	R	R
*Escherichia coli* (MDR)	8	R
*Escherichia coli* ATCC 25922	22	R
*Klebsiella pneumoniae* (CI)	21	8
*Klebsiella pneumoniae* (FI)	19	R
*Klebsiella pneumoniae* (MDR)	15	R
*Listeria innocua* (FI)	13	R
*Listeria monocytogenes* ATCC 7644	28	R
*Proteus vulgaris* (MDR)	11	9
*Providencia rustigianii* (MDR)	16	25
*Pseudomonas aeruginosa* DSM 50071	15	11
*Pseudomonas fluorescens* P1	13	R
*Salmonella enteritidis* ATCC 13076	21	8
*Salmonella infantis* (FI)	17	R
*Salmonella kentucky* (FI)	12	R
*Salmonella typhimurium* SL 1344	24	R
*Serratia odorifera* (MDR)	7	R
*Shigella boydi* (CI)	28	R
*Shigella flexneri* (CI)	13	R
*Shigella flexneri* RSKK 184	18	R
*Staphylococcus aureus* (CI)	30	18
*Staphylococcus aureus* 2 (CI)	21	R
*Staphylococcus aureus* ATCC 25923	21	R
*Staphylococcus aureus* MRSA	R	45
*Staphylococcus aureus* MRSA 2	22	38
*Staphylococcus epidermidis* DSM 20044	22	24
*Staphylococcus haemolyticus* (CI)	12	R
*Staphylococcus hominis* (CI)	24	9
*Staphylococcus hominis* ATCC 27844	18	35
*Staphylococcus lugdunensis* (CI)	22	R
*Staphylococcus warneri* ATCC 27836	23	24
*Streptococcus mutans* (CI)	12	24
*Streptococcus pneumoniae* (MDR)	10	9

A dash (-) indicates that there is no effect; CI: Clinical isolate; FI: foodborne; MDR: Multi drug resistance, MRSA: Methicillin resistance *Staphylococcus aureus*. R: Resistant.
